# Food allergy and anaphylaxis – 2057. Protecting food allergic consumers and celiac patients in India requries improvements in diagnostic accuracy, patient education, food handling and labeling practices

**DOI:** 10.1186/1939-4551-6-S1-P140

**Published:** 2013-04-23

**Authors:** Richard Goodman, Ashok Gupta, Padukudru A Mahesh, Anand B Singh, Nagendra Komarla, Ronald Van Ree, EN Clare Mills, Steve L Taylor

**Affiliations:** 1 Food Allergy Research and Resource Program, University of Nebraska, Lincoln, USA; 2 SMS Medical College Jaipur, India; 3 Allergy, Asthma and Chest Centre, Mysore, India; 4 Allergy and Allergen Research, Allergy and Immunology Lab, Institute of Genomics and Integrative Biology, Delhi University Campus Delhi, Delhi, India; 5 Allergy, Bangalore Allergy Centre, Bangalore, India; 6 Experimental Immunology, Academic Medical Center, Netherlands; 7 The University of Manchester, UK; 8 Food Science & Technology, Food Allergy Research and Resource Program, University of Nebraska-Lincoln, USA

## Background

Claims of marked increases in the prevalence of food allergy (FA) and celiac disease (CD) are common in the US and EU and increasingly in India where little is known about food allergy. Studies suggest increasing trends, but often lack rigorous definition of symptoms and tests. Reliance on Skin Prick Tests (SPT) or specific IgE alone, without corroborating clinical histories may be misleading. Once diagnosed, patients with FA or CD must avoid eliciting foods, which requires accurate information of food ingredients.

## Methods

A screen of suspected pulse-allergic subjects by selected clinicians in New Delhi, Chandigarh and Mysore/Bangalore was followed by laboratory IgE-tests with pulse extracts. Case histories of FA and CD from a medical college Pediatric clinic in Jaipur were reviewed. A systematic home survey conducted in Bangalore and Mysore involved more than 30,000 subjects with questionnaires and detailed follow-up with serology and SPT as part of Europrevall. A non-scientific survey of Indian food recipes and ingredients was used to consider terminology.

## Results

Based on limited data, the perceived rate of FA and CD in India by patients and clinicians is highly variable. Lack of standardized criteria, low availability and high costs of quality SPT reagents and laboratory tests (for CD and FA) hinder accurate diagnosis. Diverse terms and recipes for foods in India increases complexity. Allergy to milk and eggs is relatively common as expected. Reports of allergy to unlikely sources (e.g. brinjal, fruits and rice) are common, but are likely due to intolerance or too reliance on SPT or specific IgE binding, which can be misleading without clear clinical histories. Rare cases of severe anaphylaxis to *Vigna sp.*(blackgram, mung bean and cowpea) and groundnut were found.

## Conclusions

Preliminary evidence demonstrates that severe food allergy is present in India where dietary habits, production and use of packaged foods are changing rapidly. Based on experiences in other countries it seems appropriate to expand education and training programs for clinicians, encourage development of valid testing systems and gather reliable information to aid the food industry and government regulators develop methodsthat help the food industry protect FA and CD patients from unintended exposure.

